# WT1 Dendritic Cell Vaccine Therapy Improves Immune Profile and Prolongs Progression-Free Survival in End-Stage Lung Cancer

**DOI:** 10.7759/cureus.47320

**Published:** 2023-10-19

**Authors:** Hisashi Nagai, Ryusuke Karube

**Affiliations:** 1 Oncology, Ginza Phoenix Clinic, Tokyo, JPN; 2 Graduate School of Human and Environmental Studies, Tokai University, Tokyo, JPN

**Keywords:** wt1, progression-free survival, chemotherapy response, cancer immunotherapy, immune profile, metastatic non-small cell lung cancer, dendritic cell

## Abstract

WT1-pulsed dendritic cell (WT1-DC) therapy was performed for end-stage squamous cell lung cancer that rapidly worsened soon after completion of carboplatin and paclitaxel. A rapid improvement in immune profile was observed with the initiation of WT1-DC. Docetaxel and ramucirumab were initiated as second-line agents during WT1-DC. The improvement of the immune profile status continued, and at the same time, the cancer showed a predominant shrinkage. Progression-free survival was over 577 days, and the patient was able to lead a normal daily life with a performance status of 1. These findings suggest that WT1-DC improves the immune profile, and this may contribute to the long-lasting and sustained effect of chemotherapy.

## Introduction

In recent years, with the frequent use of immune checkpoint inhibitors in cancer therapy, anti-cancer immune function has come to occupy an important position in cancer treatment. Although there is still no method to accurately assess the level of anti-cancer immune function, prognosis can be inferred from blood cell counts and blood cell percentages in routine blood tests.

A poor immune profile status (IPS), i.e., high blood leukocyte counts, high neutrophil percentage, low lymphocyte percentage, and high neutrophil/lymphocyte (N/L) ratio, is known to be an independent poor prognostic factor in advanced cancer [1,2]. However, there are no reports on the prognostic impact of IPS on cancer response to chemotherapy.

WT1 (Wilms tumor 1) is a common cancer antigen that has been shown to be expressed in many types of cancers. WT1-pulsed dendritic cell (WT1-DC) vaccine is one of the anti-cancer immune cell therapies using the patient’s own monocytes with ex vivo culture and differentiation. WT1-DC has been reported to be successful in advanced cancer. However, there are no reports on the effect of WT1-DC on the IPS or on the effect of its combination with chemotherapy [3,4].

Here, we report a case of a patient with recurrent and multiple systemic metastases of lung cancer who received a second line of chemotherapy in combination with WT1-DC and sustained a marked response for a long time.

## Case presentation

**Figure 1:**
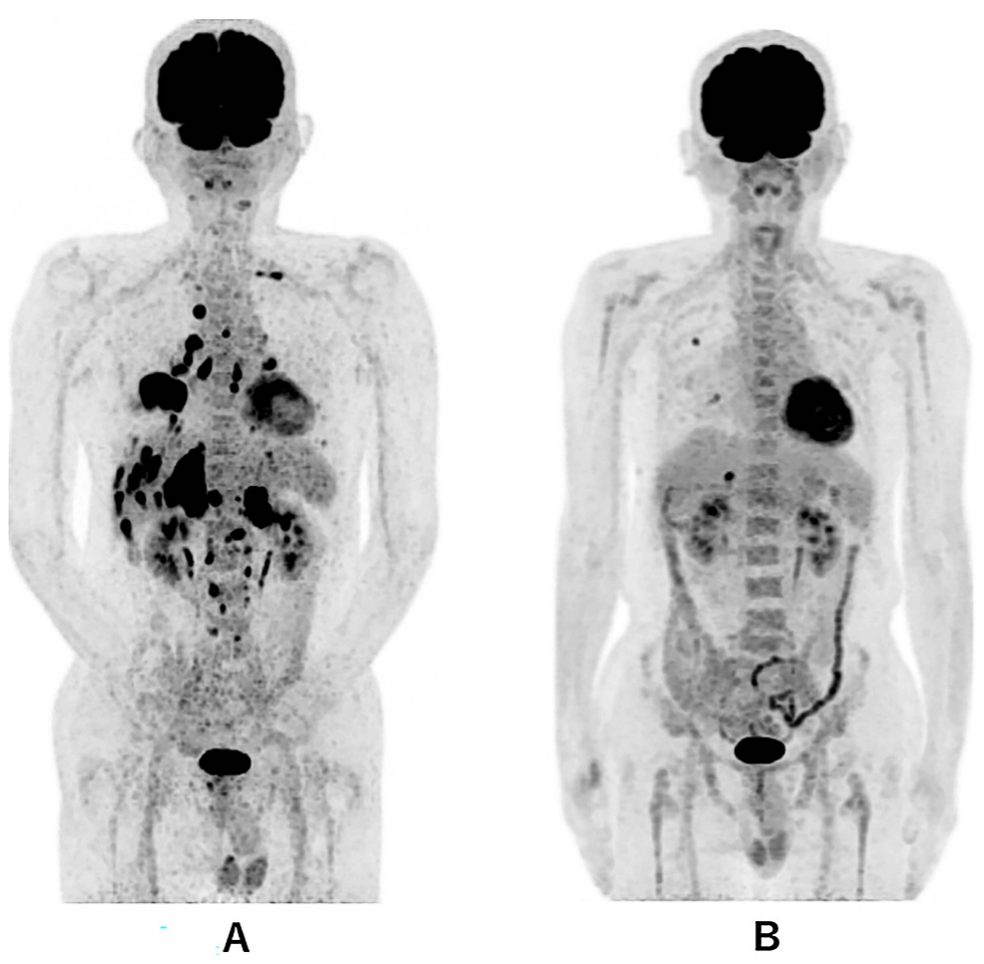
Whole-body PET-CT A: PET-CT at diagnosis showing multiple bilateral intrapulmonary metastases, multiple liver metastases, bilateral adrenal metastases, and multiple bone metastases, in addition to the primary tumor in the right lower lung. B: Whole-body PET-CT on day 479, showing two small metastases in the right lung and one in the liver.

**Figure 2:**
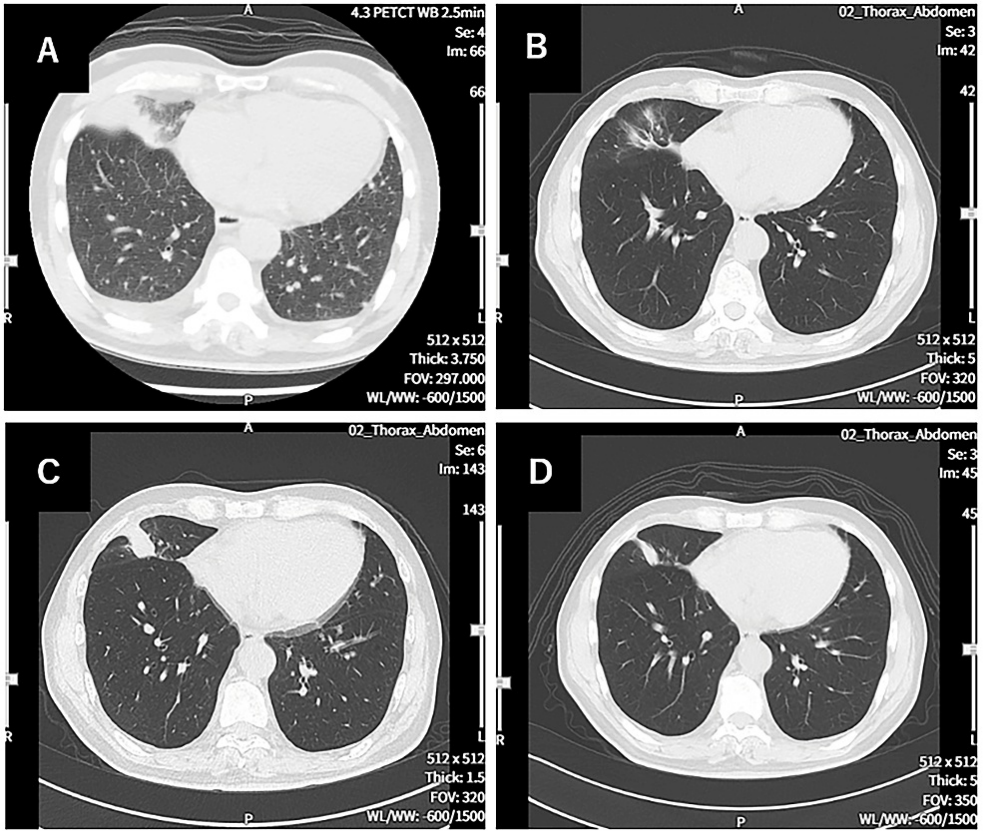
CT image of the chest A: CT image at diagnosis. Right and left pleural effusions were seen, along with the primary right lower lung cancer. B: CT image on the 114th day. The primary lesion has almost disappeared. C: CT image on the 213th day. The recurrence of the primary lesion was shown. D: CT image on the 338th day. The primary recurrent lesion has shrunken.

**Figure 3:**
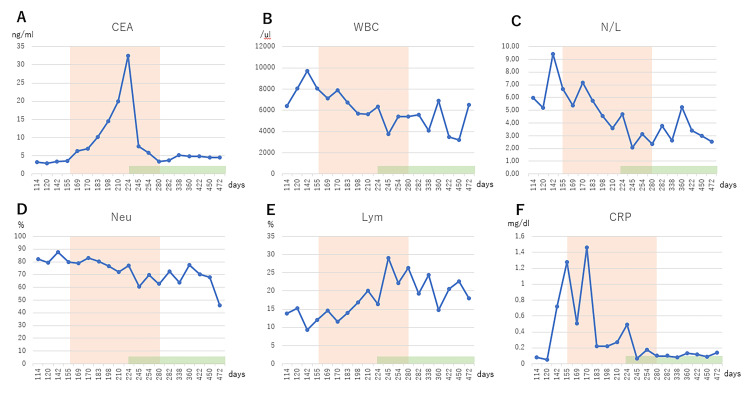
Hematological examinations Changes in hematological index from the 114th to the 472nd day of treatment. The orange area shows the duration of WT1 dendritic cell vaccine therapy. The green area shows the duration of docetaxel and ramucirumab. CEA: carcinoembryonic antigen; WBC: white blood cells; N/L: neutrophil/lymphocyte ratio; Neu: neutrophil; Lym: lymphocyte; CRP: C-reactive protein.

A 69-year-old man was diagnosed with stage IV squamous cell carcinoma of the middle lobe of the right lung, bilateral adrenal metastases, multiple liver metastases, and multiple bone metastases (Figure 1). Surgery and radiotherapy were not possible according to the guidelines, and carboplatin (CBDCA) AUC6 and paclitaxel (PTX) 200 mg/m^2^ were administered every three weeks for four cycles (until the 84th day from the first diagnosis). As a result, a clear shrinkage of the cancer was observed on chest CT on the 114th day compared to chest CT at the beginning of treatment (day 0) (Figure 2). The carcinoembryonic antigen (CEA) at diagnosis was 66.4 but decreased to 3.0 on the 121st day. However, the N/L ratio on the 121st day was high at 5.18, with a high neutrophil ratio (79.2%) and a low lymphocyte ratio (15.3%). Thereafter, the N/L ratio remained even higher, from 6.67 to 9.40, until the 155th day, during which time the CEA increased to 3.6 (Figure 3).

For concomitant immunological treatment, WT1-DC was started every two weeks starting on day 155. Monocytes collected from the patient's peripheral blood were differentiated into mature dendritic cells in a cell culture laboratory. During the culture of the dendritic cells, they were pulsed with WT1 antigen for antigen recognition. An average of 1.10 × 10^7^ mature dendritic cells was injected subcutaneously into near the left and right inguinal lymph nodes, respectively.

WT1-dendritic cell vaccine therapy was started on day 155, and a total of eight doses were administered until day 280. The intensity of cellular immune response to WT1 antigen was determined by measuring the size of delayed-type hypersensitivity (DTH). To measure DTH, 0.1 ml of WT1 antigen was injected intradermally into the center of the palmar side of the forearm. This injection was made on the same day as the inguinal administration of WT1-DC. The average of the long and short diameters of the skin erythema was calculated. The average diameter of DTH from the fourth to the eighth injection was 74.3 mm. The mean diameter of DTH did not reach 60 mm until the third injection, but from the fourth injection onward, it was above 60 mm, and the mean DTH after the fourth to the eighth injection was 74.3 mm.

On the other hand, a high fever of 38.5℃ was observed at the 7th injection, but otherwise, the fever was in the 37℃ range (Table 1). No other adverse events were observed. Immediately after the start of WT1-DC, a decrease in total white blood cell count, a decrease in neutrophil percentage, an increase in lymphocyte percentage, a decrease in N/L ratio, and a decrease in C-reactive protein (CRP) were observed. However, CEA continued to increase until after the 5th dose of WT1-DC, and chest CT on day 213 showed recurrence of the primary lung cancer lesion (Figure 2C), and CEA was as high as 32.5 on day 244 (Figure 3A). The period coinciding with the rapid rise in CEA also showed elevated CRP levels (Figure 3F).

**Table 1:** Body temperature and DTH after administration of WT1-DC Body temperature on the day after the first through the eighth doses and the mean of the long and short diameters of DTH within three days after injection. WT1-DC: WT1-pulsed dendritic cell; DTH: delayed-type hypersensitivity.

WT1-DC	1	2	3	4	5	6	7	8
Body temperature (℃)	37.1	37.4	36.7	36.8	37.1	36.8	38.5	36.9
DTH (mm)	29	35	42.5	62.5	91.5	80	72.5	65

Due to the evidence of re-exacerbation of cancer and persistently elevated CEA, the anticancer physician started docetaxel (DTX) 60 mg/m^2^ and ramucirumab 10 mg/kg every three weeks starting on day 224. As a result, the CEA was markedly reduced to 5.9 and the tumor shrunk on the 254th day. The IPS continued to improve after starting DTX and ramucirumab, with a decreased N/L ratio and, conversely, an increased lymphocyte percentage. Favorable IPS was maintained after the completion of eight doses of WT1-DC on day 280, and the same condition continued as of day 577, with no significant increase in CEA during this period. The N/L ratio from day 254 to day 577 remained low, averaging 3.27, and the lymphocyte percentage remained relatively favorable, averaging 21.0% (Figure 3).

PET-CT on the 479th day showed no abnormal findings other than two metastatic lesions with 1.0 cm in diameter in the right lung and one metastatic lesion with 1.7 cm in diameter in the liver (Figure 1B). The patient's clinical condition is good and he can lead a normal daily life with performance status 1.

## Discussion

Stage IV squamous cell carcinoma of the lung has a poor prognosis with a five-year survival rate of less than 5% [5]. Surgery and radiotherapy are not recommended in stage IV. Only chemotherapy is performed. However, second-line docetaxel and ramucirumab combination therapy is not satisfactory, with a progression-free survival period of 4.5 months and an overall survival period of 10.5 months [6]. In this case, progression-free survival has already reached 12 months, the cancer has not recurred or worsened, and the patient is clinically asymptomatic and able to lead a daily life. One possible reason for this is the concomitant use of immune cell therapy WT1-DC.

Previous studies have shown that prognosis is independently correlated with neutrophil percentage, lymphocyte percentage, and N/L ratio in many cancers, including gastric, renal, colorectal, and hepatocellular cancers [1,2,7,8]. We call these immune conditions “immune profile status.” In general, a high white blood cell count, high neutrophil percentage, low lymphocyte percentage, and high N/L ratio in advanced cancer indicate a poor prognosis. These are bad IPS. Neutrophils have recently been shown to promote cancer cell proliferation via neutrophil elastase, promote angiogenesis via secretion of vascular endothelial growth factor (VEGF) and BV8, or attenuate the anti-cancer immune response of CD8+ lymphocytes via secretion of transforming growth factor beta (TGFβ) and iNOS [9,10].

In this case, the patient was treated with CBDCA and PTX after diagnosis, and the tumor shrunk and CEA decreased. However, the white blood cell count was high, the neutrophil percentage was high, the lymphocyte percentage was low, and the N/L ratio remained high. The persistence of this poor IPS, despite the shrinking tumor and decreased CEA, represents a poor prognosis. In this case, the cancer that had shrunk with first-line chemotherapy resumed growth after the completion of four courses of treatment, with a concomitant rapid increase in CEA. Therefore, even if chemotherapy is successful, if the IPS is bad, there is a high possibility of relapse, and either continuation of chemotherapy or combination therapy to improve the immune profile should be considered.

WT1 is a common cancer antigen that is widely expressed in cancers, and the expression rate of WT1 in squamous cell lung cancer is reported to be over 90% [11]. The National Cancer Institute in the US reported that WT1 is the best in terms of expression ratio, immunogenicity, and clinical efficacy in cancer [12]. Therefore, WT1 is commonly used as a common antigen for dendritic cell vaccine therapy in Japan in clinical practice.

DTH is often used as an indicator of the therapeutic efficacy of WT1-DC, and when WT1-specific cytotoxic T cells are induced by administration of WT1 dendritic cells, an erythematous reaction appears within 48 hours after intradermal injection of WT1 antigen [4,13].

In this case, DTH with a diameter of 29 mm was identified from the first injection, and the diameter of DTH increased in proportion to increasing the number of injections. After the fourth injection, the diameter was maintained above 60 mm, indicating that sufficient WT1-specific cytotoxic T-cell induction was achieved.

WT1-DC has been reported to reduce tumor size, lower tumor markers, and extend the overall survival period [14,15]. Cancer immunotherapy, including immune checkpoint inhibitors, has been shown to improve not only response rates but also long-term survival, known as a “tail plateau shape” in the survival curve [16]. However, there are no reports on the impact of WT1-DC on the IPS. In this case, the neutrophil percentage decreased, the lymphocyte percentage increased, and the N/L ratio decreased with the initiation of the WT1 dendritic cell vaccine. The sudden increase in CRP was no longer observed and stabilized at a low level. This improvement in IPS is fundamentally important to inhibit cancer progression and improve prognosis. Therefore, the improvement of IPS should be focused on as a new feature of WT1-DC.

It has been recently reported that anticancer immunity is involved in the efficacy of anticancer drugs [17,18]. Molecularly targeted drugs such as trastuzumab, cetuximab, and panitumumab have been reported to have antibody-dependent cell-mediated cytotoxicity (ADCC) activity, and the enhanced ability of effector cells such as natural killer (NK) cells and activated lymphocytes to attack cancer cells may contribute to cancer shrinkage [19,20]. After the IPS was improved by WT1-DC in this case, the favorable IPS was maintained after starting DTX and ramucirumab, and the tumor shrinkage and maintenance have been maintained for a long time. Ramucirumab is an anti-VEGFR2 (vascular endothelial growth factor receptor 2) human monoclonal antibody, and to date there are no ADCC reports on ramucirumab. However, dendritic cells are known to interact not only with activated lymphocytes but also with NK cells. There is a possibility that ADCC may be involved in the interaction of dendritic cells with ramucirumab and activated lymphocytes or NK cells. To confirm this hypothesis, further studies are needed.

## Conclusions

Considering the above, it is needed to pay attention to the IPS when administering chemotherapy. Even if chemotherapy is successful in making the tumor size smaller, and there are no lesions observed on imaging tests such as CT and MRI, there is still a possibility of recurrence if the IPS is in poor condition. In this regard, IPS is an important prognostic factor for cancer and should continue to be monitored in daily practice. One way to improve the response rate to chemotherapy is to improve the IPS. WT1-DC may improve the immune profile and inhibit cancer progression by activating cancer-specific CD8^+^ cells and maybe NK cells. The combination of WT1-DC with molecular-targeted drugs may induce ADCC, prevent cancer recurrence, and improve long-term prognosis. Such combination therapy of immuno-cell therapy and molecular targeted drugs may be a new treatment method to improve the efficacy of conventional chemotherapy.

The relationship between immunity and cancer has attracted an extremely high level of interest in recent years. The impact of WT1-DC therapy on IPS and the relationship between IPS and the effect of chemotherapy may be significant in clarifying the relationship between immunity and cancer, and further research is expected in the future.
